# Extracellular volume-based scoring system for tracking tumor progression in pancreatic cancer patients receiving intraoperative radiotherapy

**DOI:** 10.1186/s13244-024-01689-6

**Published:** 2024-05-12

**Authors:** Wei Cai, Yongjian Zhu, Ze Teng, Dengfeng Li, Rong Cong, Zhaowei Chen, Xiaohong Ma, Xinming Zhao

**Affiliations:** Department of Diagnostic Radiology, National Cancer Center/National Clinical Research Center for Cancer/Cancer Hospital, Chinese Academy of Medical Sciences and Peking Union Medical College, Beijing, China

**Keywords:** Locally advanced pancreatic cancer, Computed tomography, Carbohydrate antigen 19-9, Intraoperative radiotherapy, Prognosis

## Abstract

**Objectives:**

To investigate the value of extracellular volume (ECV) derived from portal-venous phase (PVP) in predicting prognosis in locally advanced pancreatic cancer (LAPC) patients receiving intraoperative radiotherapy (IORT) with initial stable disease (SD) and to construct a risk-scoring system based on ECV and clinical-radiological features.

**Materials and methods:**

One hundred and three patients with LAPC who received IORT demonstrating SD were enrolled and underwent multiphasic contrast-enhanced CT (CECT) before and after IORT. ECV maps were generated from unenhanced and PVP CT images. Clinical and CT imaging features were analyzed. The independent predictors of progression-free survival (PFS) determined by multivariate Cox regression model were used to construct the risk-scoring system. Time-dependent receiver operating characteristic (ROC) curve analysis and the Kaplan–Meier method were used to evaluate the predictive performance of the scoring system.

**Results:**

Multivariable analysis revealed that ECV, rim-enhancement, peripancreatic fat infiltration, and carbohydrate antigen 19-9 (CA19-9) response were significant predictors of PFS (all *p* < 0.05). Time-dependent ROC of the risk-scoring system showed a satisfactory predictive performance for disease progression with area under the curve (AUC) all above 0.70. High-risk patients (risk score ≥ 2) progress significantly faster than low-risk patients (risk score < 2) (*p* < 0.001).

**Conclusion:**

ECV derived from PVP of conventional CECT was an independent predictor for progression in LAPC patients assessed as SD after IORT. The scoring system integrating ECV, radiological features, and CA19-9 response can be used as a practical tool for stratifying prognosis in these patients, assisting clinicians in developing an appropriate treatment approach.

**Critical relevance statement:**

The scoring system integrating ECV fraction, radiological features, and CA19-9 response can track tumor progression in patients with LAPC receiving IORT, aiding clinicians in choosing individual treatment strategies and improving their prognosis.

**Key Points:**

- Predicting the progression of LAPC in patients receiving IORT is important.
- Our ECV-based scoring system can risk stratifying patients with initial SD.
- Appropriate prognostication can assist clinicians in developing appropriate treatment approaches.

**Graphical Abstract:**

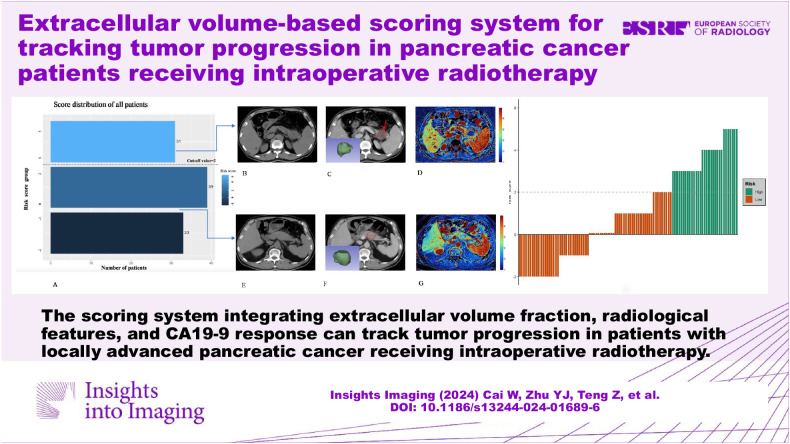

## Introduction

Approximately 30% of patients with locally advanced pancreatic cancer (LAPC) die of local progression [1], and only 11% of these patients could survive more than 3 years [2]. Therefore, improving the rate of local control and preventing local progression are crucial issues to be addressed [1, 3].

Intraoperative radiotherapy (IORT), a precise therapeutic approach that delivers high doses of radiation directly into the tumor while protecting adjacent organs, has demonstrated promising outcomes with improved local disease control rate and potential prognosis in LAPC [4]. IORT is recommended as experts’ consensus to perform on LAPC patients to relieve symptoms and obtain extra benefits [4, 5]. Around 50–80% of pancreatic ductal adenocarcinoma (PDAC) patients were assessed as stable disease (SD) based on response evaluation criteria in solid tumor version 1.1 (RECIST v.1.1) after treatment [6]. Despite no significant change in tumor diameter in these patients, some of them could achieve major pathologic response and better prognosis [7]. Some patients assessed as SD responded well and had longer progression-free survival (PFS), whereas others progressed rapidly [8]. Consequently, it is crucial to determine an efficient approach to further stratify the risk of progression in individuals assessed as SD initially after IORT. Many efforts have been made to solve this problem, including clinicopathological features, molecular biomarkers, imaging features, and radiomics [9–11]. Unfortunately, the potential of these methods is far from sufficiently investigated.

Multi-detector computed tomography is the recommended imaging technique in the evaluation of PDAC, which consists of a dual-phase contrast-enhanced protocol dedicated to the pancreas routinely [12–14]. The extracellular volume (ECV) fraction, representing the sum of the extravascular extracellular space and the intravascular space, could be estimated by means of contrast-enhanced CT (CECT) [15]. Recently, it has been revealed that ECV is associated with fibrosis and deposition of an extracellular stromal matrix of the pancreas [15, 16]. Desmoplastic stroma exhibits an essential role in tumor oncogenesis, proliferation, progression, metastasis, and chemoresistance [16, 17]. ECV fraction could be used to evaluate pancreatic fibrosis and predict tumor aggressiveness, treatment response, and prognosis in PDAC [15, 18]. Based on the above, we hypothesize that ECV might become a potential imaging biomarker for stratifying the risk of progression in SD patients. However, until recently, ECV was estimated mainly via the delayed or equilibrium phase of CECT, which prolonged examination time and limited its clinical practicality. Dual-energy CT was also employed in other investigations, but this requires specialized equipment and scanning protocols. Nevertheless, it has not been determined whether the ECV, which is derived from the portal-venous phase (PVP) of conventional CT, could be a more acceptable non-invasive imaging biomarker for prognostic prediction in PDAC. Many studies have shown that clinical factors and CT imaging features, for example, CA19-9, necrosis, and peripancreatic tumor infiltration, were valuable in predicting the prognosis of PDAC [9, 12].

A risk-scoring system could serve as a simple and efficient way to evaluate clinicopathological features or prognosis in PDAC [19]. Therefore, the purpose of this study was to investigate the potential role of ECV derived from PVP of conventional CT in predicting progression and construct a progression risk-scoring system based on ECV and clinical-radiological features in LAPC patients assessed as SD initially after IORT.

## Materials and methods

### Patients

**Fig. 1:**
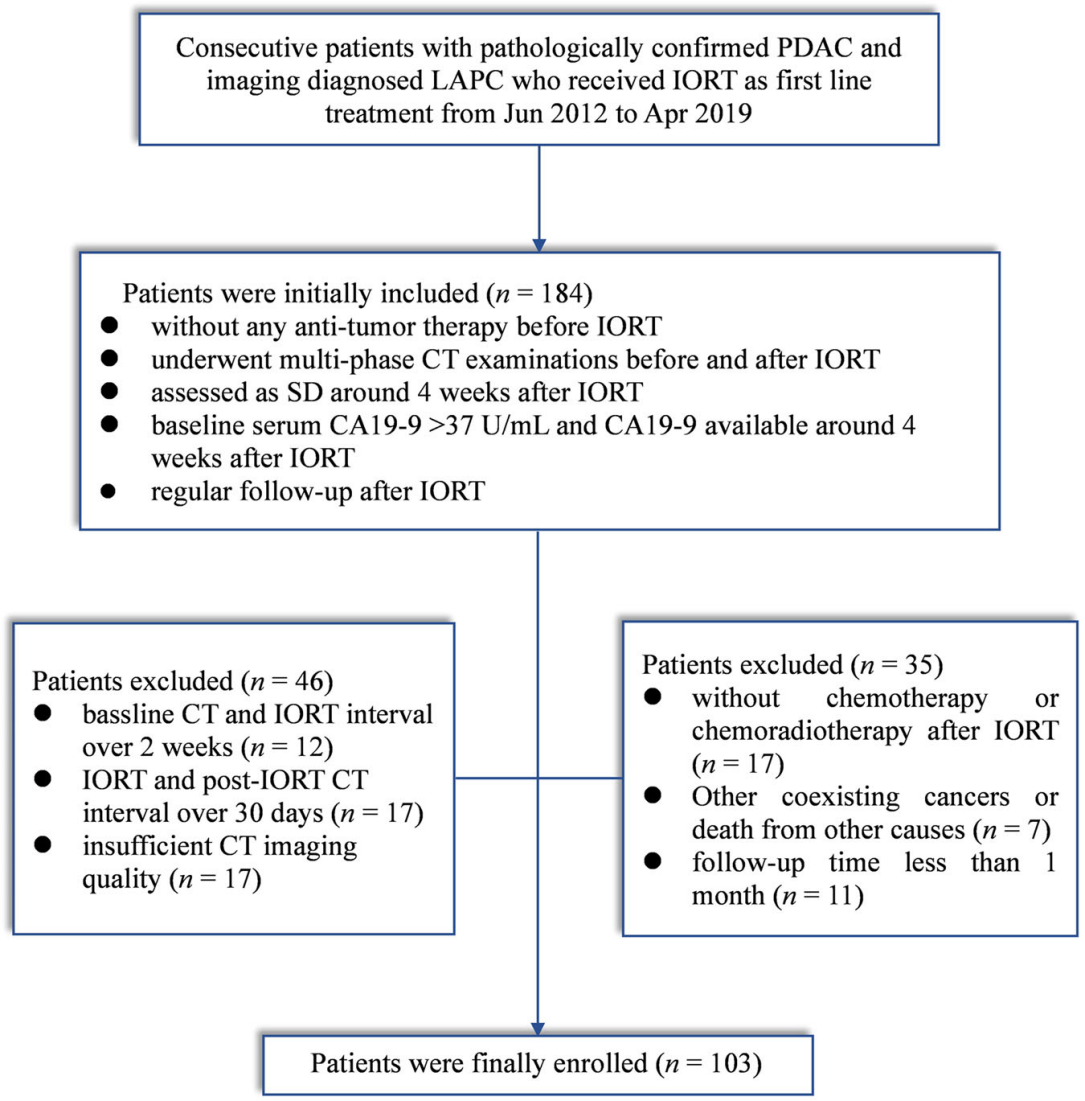
Flow chart of the patient enrollment process for patients. CA 19-9, carbohydrate antigen 19-9; LAPC, locally advanced pancreatic cancer; IORT, Intraoperative radiotherapy; pre- and post-IORT, before and after IORT

The institutional review board approved this retrospective study and waived the requirement for informed consent. Between June 2012 and April 2019, we initially enrolled 184 patients with LAPC who received IORT as first-line treatment according to multidisciplinary team discussion at our hospital. LAPC was diagnosed in accordance with National Comprehensive Cancer Network (NCCN) guidelines [14]. The inclusion criteria were as follows: (a) without any anti-tumor therapy before IORT; (b) underwent multi-phase CECT examinations before and after IORT; (c) treatment response was initially assessed as SD by RECIST v.1.1 after 4 weeks of IORT; (d) baseline serum CA19-9 > 37 U/mL and CA19-9 available at around 4 weeks after IORT; (e) regular follow-up after IORT. Eighty-one patients were excluded for certain reasons as listed in Fig. 1.

Finally, 103 patients including 64 males and 39 females (mean age, 58.52 ± 10.09 years) were recruited in this study. The flow chart of the patient enrollment process and study design are depicted in Figs. 1 and S1.

### CT technique

All patients underwent a standard multi-phase CECT dedicated to pancreas acquisition in non-enhanced (N), arterial phase (AP), pancreatic parenchymal phase (PPP), and PVP. Iopromide (Ultravist 370, Schering, Berlin, Germany) was administered at a rate of 3.5 mL/sec, with a weight-dependent dose of 1.5 mL/kg. After the contrast agent injection, the average delay times of AP, PPP, and PVP were 25–30 s, 40–50 s, and 65–70 s, respectively. Multiple CT scanners were used because of the retrospective design of this study. The detailed CT scan parameters are listed in Table S1.

### ECV fraction analysis

Registration was first performed to alleviate the negative impacts resulting from phase mismatch through an open-sourced Python package (ANTsPy, https://github.com/ANTsX/ANTsPy). The registered CT images were processed using an in-house developed program written in Python (version 3.12.0). According to the previous study [18], ECV map can be calculated as the ratio of enhancement of the tumor tissue to the enhancement of the blood pool (abdominal aorta) on PVP-enhanced CT multiplied by the difference of 1-minus the hematocrit value, using the formula (1) by a pixel-wise method employing N and PVP CT images as input:

1 $${{{{{\rm{ECV}}}}}}\,=\,\left(1\,-\,{{{{{\rm{hematocrit}}}}}}\right)\left(\frac{{\varDelta {{{{{\rm{HU}}}}}}}_{{{{{{\rm{tumor}}}}}}}}{{\varDelta {{{{{\rm{HU}}}}}}}_{{{{{{\rm{aorta}}}}}}}}\right)\,\times\,100\%$$

where ΔHU_tumor_ and ΔHU_aorta_ represent the difference in the CT attenuation of the tumor and aorta between the unenhanced and PVPs.

All images were analyzed by two abdominal radiologists (Z.T and D.L with 8 and 6 years of experience in abdominal imaging) who were blinded to the clinical information. Using 3D slicer (version 5.2.2; www.slicer.org), an open-source image processing software, the entire tumor was included in the freehand volume of interest (VOI) on PVP images slice by slice, carefully avoiding adjacent vasculature, necrosis, and cysts, since these components may affect the accuracy of the estimation of tumor ECV values. Then the VOIs were automatically copied to ECV maps to extract the mean values of ECV. The average of the measurements from the two radiologists was used for further analysis. Interobserver agreement on the segmentations was evaluated by the Dice similarity coefficient (DSC). A DSC greater than 0.80 was considered satisfactory reproducibility. Discrepancies in the segmentations (DSC < 0.50) were resolved by consensus.

### Radiological feature analysis

Two abdominal radiologists (Y.Z and W.C with 10 and 5 years of abdominal imaging experience, respectively) who were aware of the diagnosis of PDAC but blinded to the clinical details independently reviewed the CT images. Seven radiological features before IORT were evaluated, including tumor location, necrosis, rim-enhancement, peripancreatic fat infiltration, pancreatic duct dilatation, atrophic upstream pancreatic parenchyma, and suspicious lymph node metastasis. The definitions of these features were proposed by the Society of Abdominal Radiology dedicated to PDAC and previous studies (Table S2) [20–22]. Detailed steps to ensure consistency during evaluation were provided in Appendix E1. Inter-reader agreements were evaluated after independent image analysis, and all discrepancies were resolved during the second reading.

### Clinical data

Clinical data were collected from the electronic medical records, including age, sex, body mass index (BMI), adjuvant therapy, jaundice, clinical American Joint Committee on Cancer (AJCC) TNM stage [14], serum CA19-9 level at baseline and after IORT (if the patients had obstructive jaundice, resampled after biliary drainage), carcinoembryonic antigen (CEA), carbohydrate antigen 242 (CA 242), hematocrit, bilirubin, albumin, D-dimer, fibrinogen, glucose, and transferrin at baseline, which were included for the reasons listed in the Table S3.

The CA19-9 response was defined as an over 50% reduction from the baseline level or decreasing to the normal range (below 37 U/mL) after IORT. The CA19-9 change was calculated using the formula (2):

2 $${{{{{\rm{CA}}}}}}19-9\;{{{{{\rm{change}}}}}}\,=\,	 \frac{{{({{{{{\rm{post}}}}}}-{{{{{\rm{IORT}}}}}}\,CA19-9}})\,-\,\left({{{{{\rm{baseline}}}}}}\,{{{{{\rm{CA}}}}}}19-9\right)}{\left({{{{{\rm{baseline}}}}}}\,{{{{{\rm{CA}}}}}}19-9\right)}\,\\ 	 \times\,100\%$$

### IORT and adjuvant therapy

The IORT procedure and sequential adjuvant treatment regimen were determined by a standardized protocol reported in experts’ consensus [4] and established by the abdominal oncology multidisciplinary team at our institution. All patients received chemotherapy or chemoradiotherapy after IORT. Detailed information is provided in Appendix E2, Table S4, and Fig. S2.

### Follow-up

All patients were regularly followed up through outpatient clinic visits. Physical examination and laboratory tests were performed monthly. Imaging examination was performed every 3 months. The end of the follow-up date was June 30, 2019. PFS was defined as the time from IORT to any event of local tumor progression, distal metastasis, or death associated with the tumor.

### Statistical analysis

Variables were analyzed using independent sample *t* test, Mann–Whitney *U*, Wilcoxon *χ*^2^, or Fisher’s exact test as appropriate. Consistency between readers was evaluated using Cohen kappa statistics for CT radiological features and intraclass correlation coefficient for quantitative parameters (< 0.20, poor; 0.21–0.40, fair; 0.41–0.60, moderate; 0.61–0.80, good; and 0.81–1.00, excellent).

The Cox proportional hazards model was conducted to identify independent predictors for PFS. Referring to Sullivan’s method [23], a risk-scoring system was developed depending on the β coefficients of the Cox regression model. Time-dependent receiver operating characteristic curve (ROC) analysis was applied to evaluate the predictive ability of the risk-scoring system. In addition, calibration plots with 1000 bootstrap resamples were used to assess the accuracy of survival rate prediction. An outcome-based optimal cut-off value for the total risk score was determined using a maximally selected rank statistics algorithm (Maxstat package) in R statistical software (R, version 4.3.0; R Foundation for Statistical Computing, Vienna, Austria) [24]. PFS was assessed using Kaplan–Meier method and compared by log-rank test. A two-sided *p* value < 0.05 was considered statistically significant. All statistical analyses were conducted using R software.

## Results

### Patient characteristics

The demographic and clinical characteristics of the patients are presented in Table 1. The time interval from baseline CT to IORT was 10.0 days (range, 8.0–14.0 days) (Fig. S3).

**Table 1:** Baseline characteristics and CT imaging features of 103 patients and univariate Cox analysis for PFS

Characteristic	All patients^a^	Univariate Cox analysis	
	(*n* = 103)	HR (95% CI)	*p* value
Baseline characteristic			
Age (years)	58.52 ± 10.09	1.006 (0.983, 1.029)	0.630
Sex			
Male	64 (62.1)	Reference	
Female	39 (37.9)	0.987 (0.616, 1.583)	0.958
Adjuvant therapy			
Chemotherapy	65 (63.1)	Reference	
Chemoradiotherapy	38 (36.9)	1.316 (0.876, 1.975)	0.181
AJCC 8^th^ T stage			
T1-2	56 (54.4)	Reference	
T3-4	47 (45.6)	1.202 (0.761, 1.899)	0.431
AJCC 8^th^ N stage			
N0	48 (46.6)	Reference	0.831
N1	26 (25.2)	1.211 (0.653, 2.245)	0.543
N2	29 (28.2)	1.035 (0.568, 1.887)	0.910
Jaundice			
Absent	53 (51.5)	Reference	
Present	50 (48.5)	1.141 (0.720, 1.810)	0.574
BMI (kg/m^2^)	24.24 (20.55, 29.40)	1.047 (0.995, 1.101)	**0.043**
Pre-CA19-9 (U/mL)	171.90 (68.40, 789.00)	1.000 (1.000, 1.000)	0.117
Post-CA19-9 (U/mL)	73.50 (39.20, 165.30)	1.000 (1.000, 1.000)	0.102
CA19-9 response			
Present	64 (62.1)	Reference	
Absent	39 (37.9)	1.895 (1.263, 2.844)	**0.002**
CEA (ng/mL)	4.60 (3.34, 9.49)	1.017 (0.999, 1.036)	0.069
CA 242 (U/mL)	34.23 (10.20, 150.00)	1.002 (0.998, 1.006)	0.306
TBil (μmol/L)	19.30 (10.75, 157.95)	1.000 (0.998, 1.002)	0.990
DBil (μmol/L)	6.80 (4.50, 137.20)	1.001 (0.998, 1.003)	0.495
D-dimer (mg/L)	0.32 (0.19, 1.02)	1.008 (0.972, 1.046)	0.673
Fibrinogen (g/L)	3.35 ± 1.12	0.929 (0.753, 1.146)	0.491
Hematocrit (L/L)	0.43 (0.40, 0.45)	0.963 (0.768, 1.104)	0.109
Glucose (mmol/L)	6.07 (5.49, 8.22)	0.992 (0.983, 1.002)	0.115
Transferrin (mg/dL)	229.71 ± 44.25	0.999 (0.994, 1.005)	0.759
ALB (g/L)	43.80 (40.00, 46.80)	0.985 (0.941, 1.031)	0.516
CT imaging features			
Quantitative parameters			
Diameter (cm)	3.20 (2.60, 3.95)	0.959 (0.812, 1.134)	0.627
ΔHU_tumor_ (HU)	30.7 (21.24, 40.22)	0.973 (0. 901, 1.004)	**0.039**
ΔHU_tumor_/ΔHU_aorta_	92.4 (76.6, 108.2)	0.941 (0.897, 1.193)	**0.028**
ECV (%)	19.1 (14.7, 23.3)	0.927 (0.889, 0.967)	**< 0.001**
Radiological features			
Tumor Location			
Head/uncinate	71 (68.9)	Reference	
Body/tail	32 (31.1)	1.080 (0.639, 1.825)	0.773
Necrosis			
Absent	46 (44.7)	Reference	
Present	57 (55.3)	1.283 (0.721, 2.284)	0.396
Rim-enhancement			
Absent	66 (64.1)	Reference	
Present	37 (53.9)	2.011 (1.337, 3.026)	**0.001**
Peripancreatic fat infiltration			
Absent	62 (60.2)	Reference	
Present	41 (39.8)	1.816 (1.198, 2.754)	**0.005**
Suspicious lymph nodes			
Absent	55 (53.4)	Reference	
Present	48 (46.6)	0.986 (0.621, 1.565)	0.952
Pancreatic duct dilatation			
Absent	49 (47.6)	Reference	
Present	54 (52.4)	1.122 (0.711, 1.772)	0.621
Atrophic upstream pancreatic parenchyma			
Absent	46 (44.7)	Reference	
Present	57 (55.3)	1.150 (0.724, 1.827)	0.554

Variables with *p* < 0.05 in univariate analysis were highlighted in bold and applied to multivariate analysis using a stepwise Cox proportional hazards regression model

*AJCC* American Joint Committee on Cancer, *ALB* albumin, *BMI* body mass index, *CA19-9* carbohydrate antigen 19-9, *CEA* carcinoembryonic antigen, *CA 242* cancer antigen 242, *CI* confidence interval, *TBil* total bilirubin, *DBil* direct bilirubin, *ECV* tumor extracellular volume, *HR* hazard ratio, *PFS* progression-free survival

^a^Data are reported as mean ± standard deviation or median with interquartile range in parentheses for continuous variables and number (%) of patients for categoric variables

The median follow-up time was 6.90 months (range, 1.60–38.00 months). All patients developed disease progression after IORT during follow-up. The overall median PFS was 6.40 months (95% confidence interval [CI]: 4.95–7.86 months). The PFS rates at 3 months, 6 months, and 1 year were 88.3%, 52.4%, and 20.4%, respectively.

### CT imaging features assessment

The CT imaging features (radiological features and quantitative parameters) are summarized in Table 1. The DSC of the VOI segmentation was 0.84 ± 0.06. Kappa and Interobserver agreement analyses showed good or excellent agreement for radiological features (0.70–1.00) (Table S5) and CT quantification parameters (0.82–0.87) (Table S6).

### Identification of independent risk factors for PFS

**Fig. 2:**
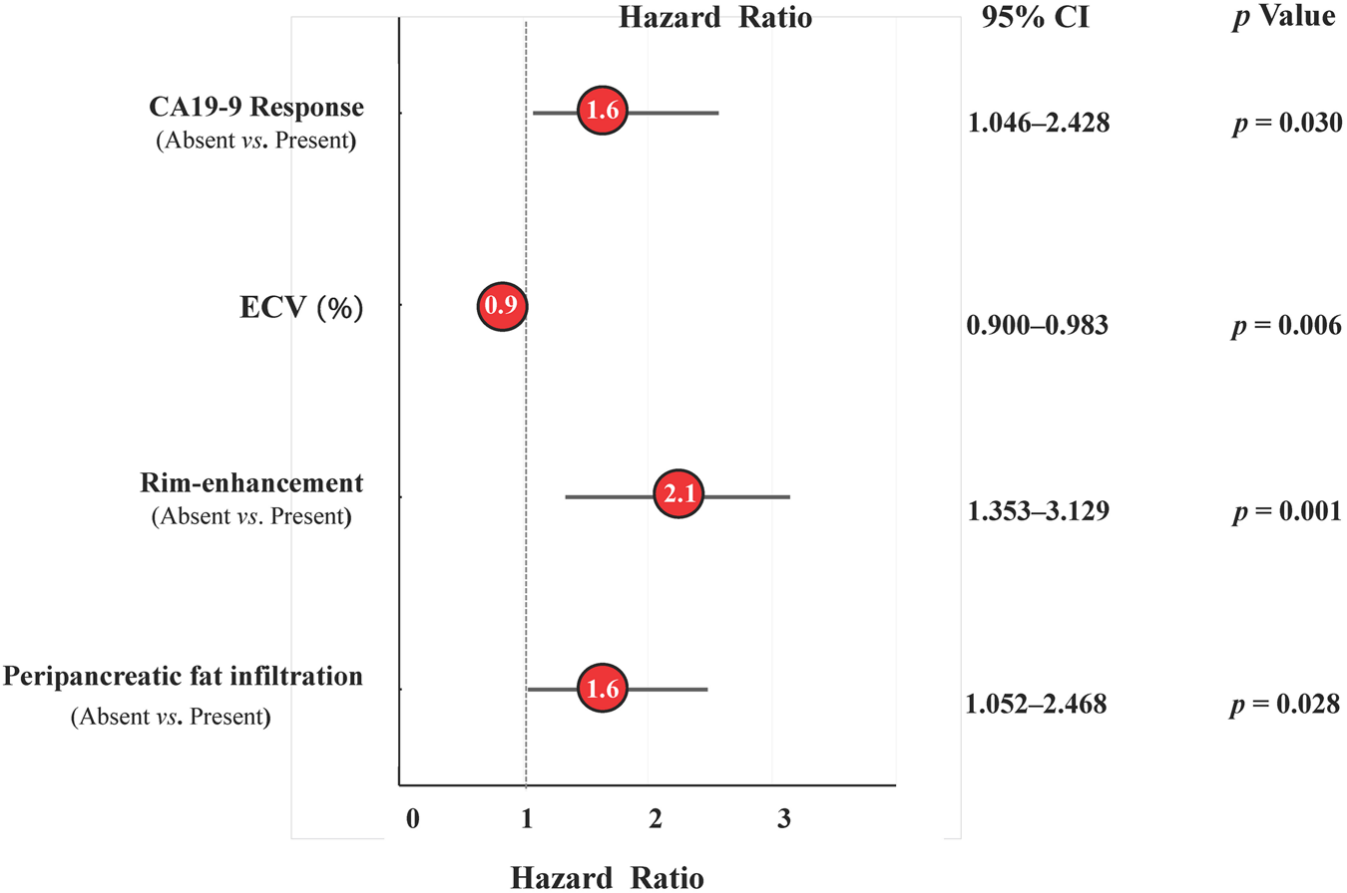
Forest plots for the multivariable Cox regression analysis for PFS. The vertical dotted line represents hazard ratios equal to 1.0. Numbers inside the red circles are HRs, and horizontal lines on either side of the circles represent 95% CIs. CA 19-9, carbohydrate antigen 19-9; CI, confidence interval; ECV, tumor extracellular volume; PFS, progression-free survival

Univariate Cox analysis identified seven factors associated with PFS in patients with LAPC receiving IORT, including BMI, CA19-9 response, ΔHU_tumor_, ΔHU_tumor_/ΔHU_aorta_, ECV, rim-enhancement, and peripancreatic fat infiltration (all *p* < 0.05) (Table 1). Through multivariate Cox analysis, the independent factors correlated with PFS were CA19-9 response (hazard ratio [HR], 1.594; 95% CI: 1.046–2.428; *p* = 0.030), ECV (HR, 0.941; 95% CI: 0.900–0.983; *p* = 0.006), rim-enhancement (HR, 2.058; 95% CI: 1.353–3.129; *p* = 0.001), and peripancreatic fat infiltration (HR, 1.612; 95% CI: 1.052–2.468; *p* = 0.028) (Table 2 and Fig. 2).

**Table 2:** Multivariate Cox proportional hazard analysis for PFS of LAPC patients

Variables	Multivariate analysis	
	HR (95% CI)	*p* value
BMI (kg/m^2^)	…	…
CA19-9 response		
Present	Reference	
Absent	1.594 (1.046, 2.428)	**0.030**
ΔHU_tumor_ (HU)	…	…
ΔHU_tumor_/ΔHU_aorta_	…	…
ECV (%)	0.941 (0.900, 0.983)	**0.006**
Rim-enhancement		
Absent	Reference	
Present	2.058 (1.353, 3.129)	**0.001**
Peripancreatic fat infiltration		
Absent	Reference	
Present	1.612 (1.052, 2.468)	**0.028**

The ellipsis indicates *p* value is not significant and should be excluded from the multivariate Cox model

Variables with *p* < 0.05 in multivariate Cox hazards regression analysis were highlighted in bold

*PFS* progression-free survival, *HR* hazard ratio, *CI* confidence interval, *CEA* carcinoembryonic antigen, *CA19-9* carbohydrate antigen 19-9, *ECV* tumor extracellular volume

### Development of risk-scoring system for PFS

**Fig. 3:**
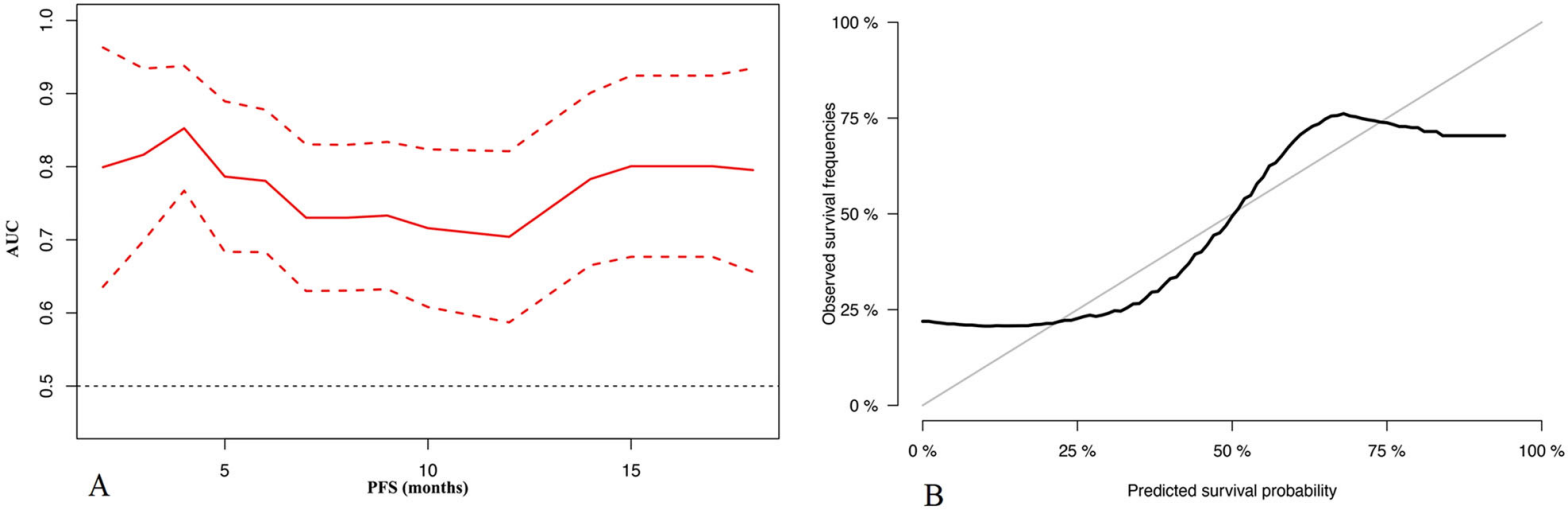
Predictive performance of risk-scoring system. **A** The time-dependent area under the ROC curve (AUC) plot with a 95% confidence interval shows the risk-scoring system in predicting PFS for each follow-up time point within 18 months. **B** Calibration plot for the overall survival prediction at the 6-month. PFS, progression-free survival; ROC, receiver operating characteristic

A risk-scoring system based on the independent risk factors for PFS was developed, with total scores ranging from −2 to 5 (Table 3). Time-dependent area under curves (AUCs) within 18 months were employed to assess the accuracy of the risk-scoring system, which demonstrated good performance with AUCs all above 0.70 (Fig. 3A). The 6-month calibration curve for prediction risk and actual observation of progression agreed satisfactory (Fig. 3B).

**Table 3:** The risk-scoring system for the prognosis prediction in LAPC patients assessed as SD

Evaluations	Scores
CA19-9 response	
Present	0
Absent	2
Rim-enhancement	
Absent	0
Present	1
ECV	
≥ 20%	−2
16–20%	0
< 16%	1
Peripancreatic fat infiltration	
Absent	0
Present	1

*CA19-9* carbohydrate antigen 19-9, *ECV* tumor extracellular volume, *SD* stable disease

### Risk stratification for predicting progression and post hoc subgroup analysis

**Fig. 4:**
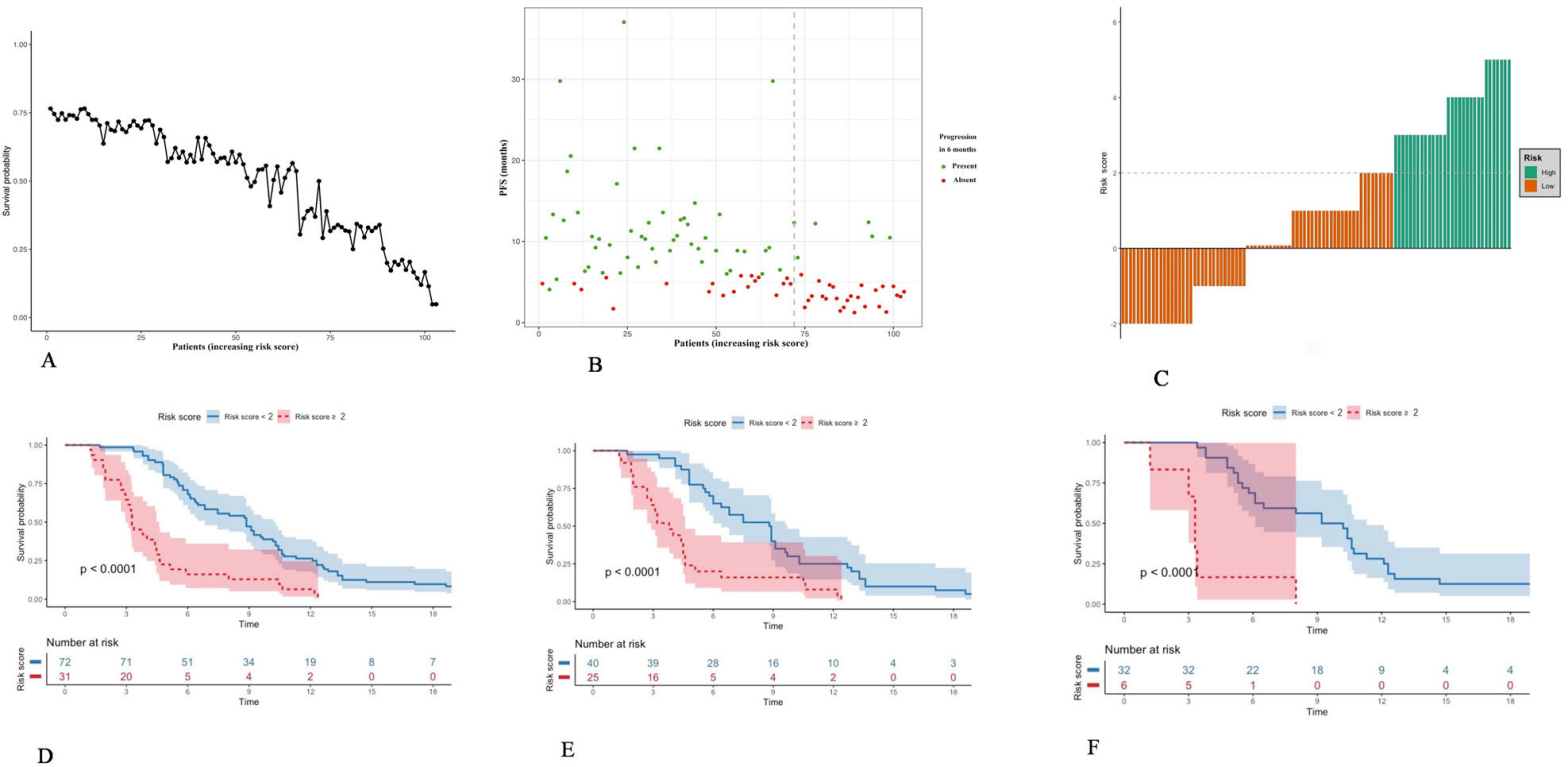
The relationship between risk score and progression. **A** The disease progression pattern plot demonstrates a decreasing probability of progression-free survival along with an increasing risk score at 6 months for LAPC patients. **B** The patients were divided into two groups according to the threshold of the cut-off value of the risk score. Green represents the low-risk group. Orange represents the high-risk group. The dashed-gray line represents the cut-off value. **C** The waterfall plot shows the distribution of the total risk score of individuals and the predicted progression risk of LAPC patients after IORT according to the scoring system. The dashed-gray line represents the cut-off value. **D** Kaplan–Meier curves show the overall survival of patients in the high-risk and low-risk groups. **E**, **F** Kaplan–Meier PFS curves in post hoc subgroup analyses according to postoperative adjuvant chemotherapy or chemoradiotherapy. IORT, Intraoperative radiotherapy; LAPC, locally advanced pancreatic cancer; PFS, progression-free survival

**Fig. 5:**
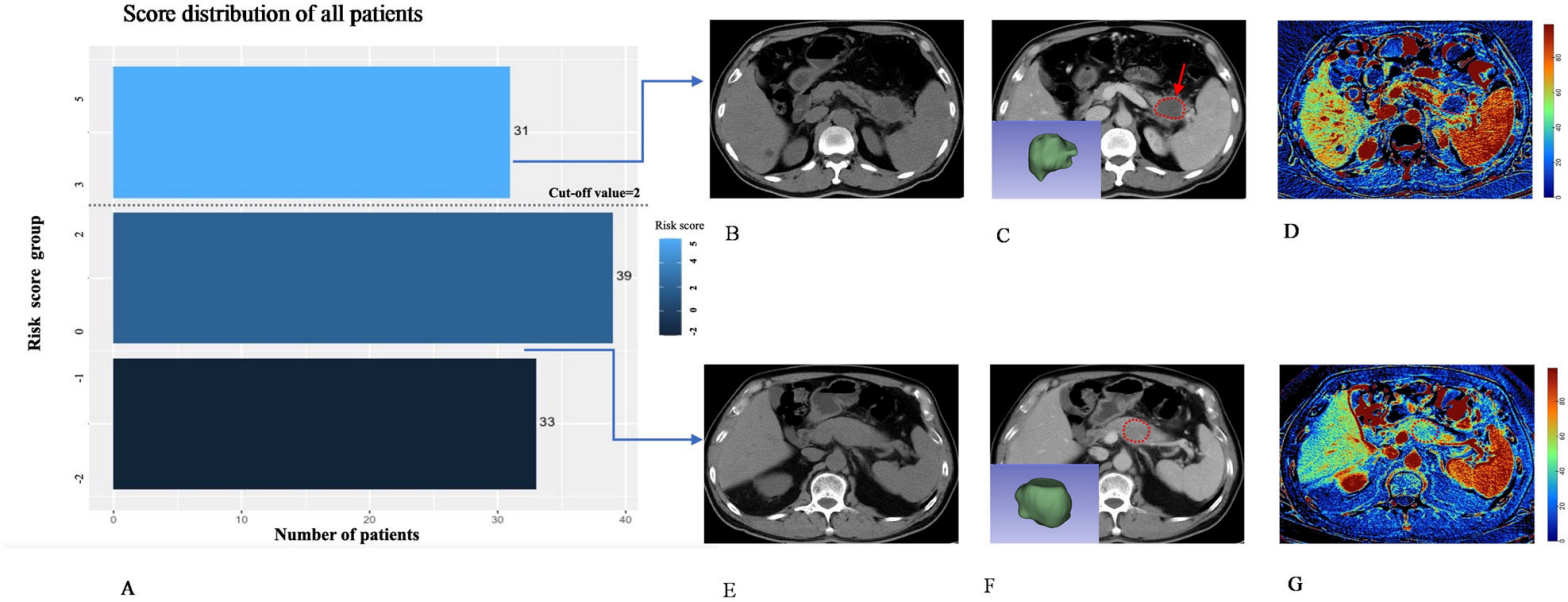
Distribution of risk scores and examples of groups at different risks of progression. **A** Histogram of the distribution of risk score is shown on the left. The dashed line represents the cut-off value. The right side of the figure shows examples of CT imaging features in different risk groups. From left to right, the images of non-enhanced (**B**, **E**), PVP with VOI of tumor at the lower left (**C,** **F**), and ECV map (**D**, **G**) are shown sequentially. **B**–**D** A 64-year-old man was diagnosed as LAPC with a 4.3 cm tumor at the tail of the pancreas before IORT and was assessed as SD after IORT. The patient presented rim-enhancement and peripancreatic fat infiltration (arrow, **C**) with an ECV fraction of 15.9%, and CA19-9 non-response. He was predicted at high risk of progression with a total risk score of 5 points. Finally, this patient progressed due to liver metastases at 3.5 months. **E**–**G** A 61-year-old man with LAPC at the body of the pancreas before IORT. In this case, rim-enhancement and peripancreatic fat infiltration were absent (**F**), while the ECV fraction was 23.7% and CA19-9 was grouped as a response. Based on a total risk score of −2 points, this patient was classified as low risk of progression. After 16.5 months of IORT, the patient had local progression. CA 19-9, carbohydrate antigen 19-9; ECV, extracellular volume; IORT, intraoperative radiotherapy; LAPC, locally advanced pancreatic cancer; SD, stable disease; VOI, volume of interest; PVP, portal-venous phase

For LAPC patients, the probability of PFS at 6 months, dropped as the total risk score increased (Fig. 4A). Furthermore, a higher score was associated with poorer outcomes for LAPC (Fig. 4B). A cut-off value of 2 points was determined to stratify patients into high-risk (≥ 2 points) (*n* = 31) and low-risk (< 2 points) (*n* = 72) for progression (Fig. 4C). Patients with low-risk progressed slower than those with high-risk after IORT, with median PFS of 8.90 months (95% CI: 7.48–10.31 months) and 4.40 months (95% CI: 3.55–5.25 months), respectively (*p* < 0.001) (Table 4 and Fig. 4D). The 3-, 6- and 12-month PFS rates in the low-risk group were 93.1%, 72.2%, and 25.0%, while 81.0%, 12.9%, and 0.6%, respectively, in the high-risk group (all *p* < 0.001) (Table 4). The distribution of risk scores and representative cases for different risk groups were shown in Fig. 5. The predictive ability for progression as estimated using the risk-scoring system at various time points was summarized in Table 5 and Fig. S4. Notably, the risk-scoring system demonstrated the highest efficacy in predicting ≥ 30% probability of 1-year PFS, with an accuracy of 94.4%.

**Table 4:** Analysis for PFS between predicted high- or low-risk group

	Low-risk, (*n* = 72)	High-risk, (*n* = 31)	*p* value
Median PFS (months) (95% CI)^#^	8.90 (7.48, 10.31)	4.40 (3.55, 5.25)	< 0.001
PFS rate in 3 months^a^	*n* = 67, 93.1%	*n* = 25, 81.0%	< 0.001
PFS rate in 6 months^a^	*n* = 52, 72.2%	*n* = 4, 12.9%	< 0.001
PFS rate in 12 months^a^	*n* = 18, 25.0%	*n* = 3, 9.6%	< 0.001

*PFS* progression-free survival, *CI* confidence interval

^#^*p* Value was calculated by log-rank tests

^a^Data are reported as the number and percentage of patients, and *p* value was calculated by χ2 tests

**Table 5:** Prediction performance for PFS of risk score

Time	PFS probability	Accuracy (%)	Sensitivity (%)	Specificity (%)	PPV (%)	NPV (%)
3 months	≥ 90%	60.2 (50.1–69.7) [62/103]	56.0 (45.2–66.4) [51/91]	91.7 (61.5–99.8) [11/12]	98.1 (89.7–99.9) [51/52]	21.6 (11.3–35.3) [11/51]
	≥ 75%	83.5 (74.9–83.5) [86/103]	93.4 (86.2–97.5) [85/91]	8.3 (0.2–38.5) [1/12]	88.5 (80.4–94.1) [85/96]	14.3 (0.4–57.9) [1/7]
6 months	≥ 60%	75.7 (66.3–83.6) [78/103]	73.1 (59.0–84.8) [38/52]	78.4 (64.7–88.7) [40/51]	79.6 (63.3–88.2) [38/49]	74.1 (60.3–85.0) [40/54]
	≥ 40%	70.9 (61.1–79.4) [73/103]	80.6 (62.5–92.5) [25/31]	66.7 (54.6–77.3) [48/72]	51.0 (36.3–65.6) [25/49]	88.9 (77.4–95.8) [48/54]
	≥ 20%	58.3 (48.1–67.9) [60/103]	75.0 (42.8–94.5) [9/12]	56.0 (45.2–66.4) [51/91]	18.4 (8.8–32.0) [9/49]	94.4 (84.6–98.8) [51/54]
12 months	≥ 30%	94.4 (84.6–98.8) [70/103]	83.6 (73.3–91.2) [61/73]	33.3 (14.7–49.4) [9/30]	77.6 (63.3–88.2) [61/82]	42.9 (21.8–66.0) [9/21]
	≥ 20%	62.1 (52.0–71.5) [64/103]	92.2 (97.8–81.1) [47/51]	53.1 (34.7–70.9) [17/32]	57.3 (45.9–68.2) [47/82]	81.0 (58.1–94.6) [17/21]
	≥ 10%	46.6 (36.7–56.7) [48/103]	85.7 (57.2–98.2) [29/31]	26.4 (16.7–38.1) [19/72]	35.4 (25.1–46.7) [29/82]	90.1 (69.6–98.8) [19/21]

Data are percentages with 95% CIs in parentheses and numbers of observations in brackets

*PFS* progression-free survival, *CI* confidence interval, *PPV* positive predictive value, *NPV* negative predictive value

Post-hoc subgroup analysis showed significant prognostic differences (all *p* < 0.001) between low- and high-risk patients in patients either receiving adjuvant chemotherapy or chemoradiotherapy postoperatively (Fig. 4E, F).

### Serial tumor response after IORT

**Fig. 6:**
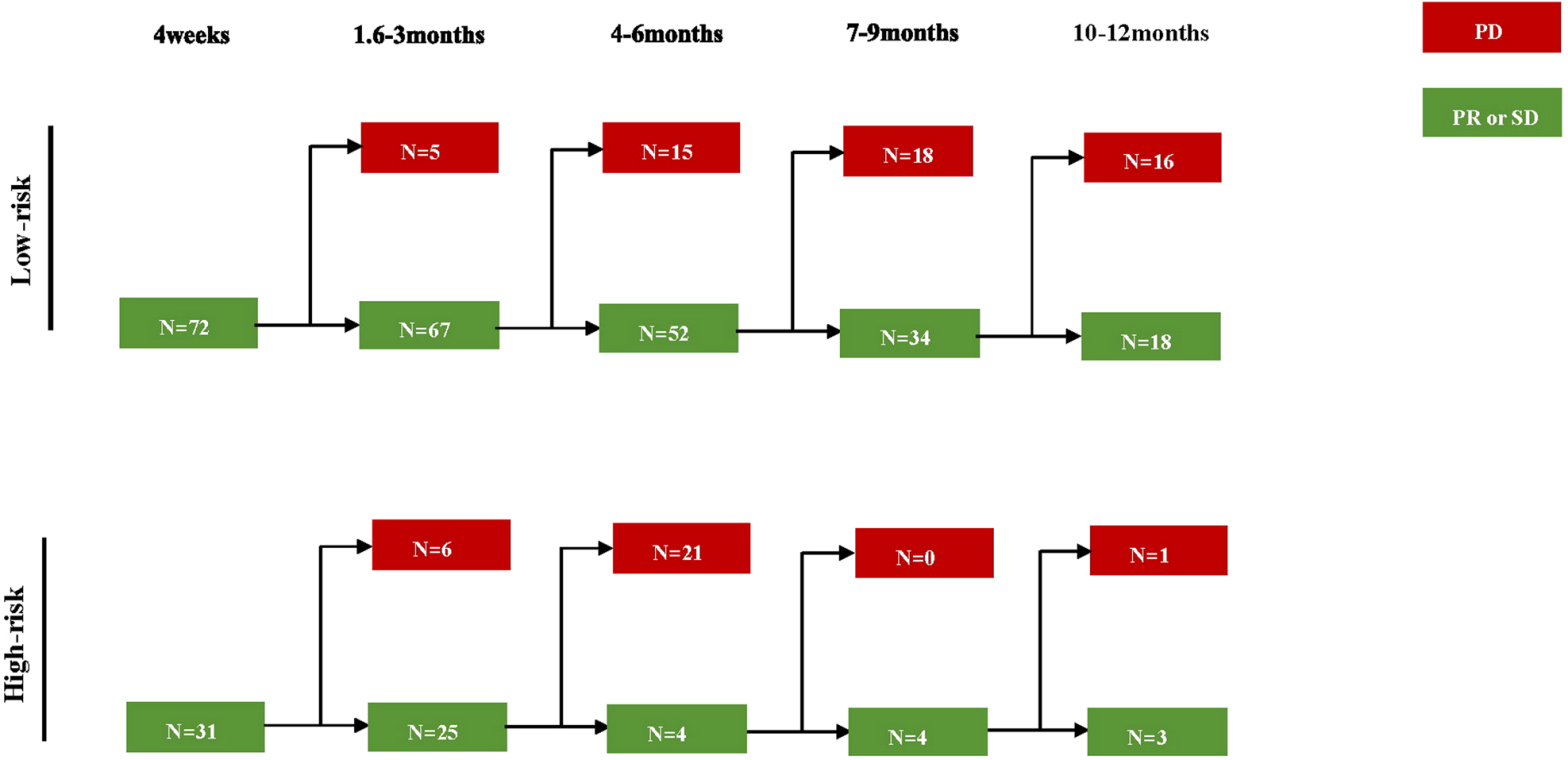
Serial tumor progression after treatment according to risk stratification in SD patients. The diagram recorded the outcome of progression at 3 months, 6 months, 9 months, and 12 months, respectively. N the number of patients; PD, progressive disease; PR, partial response; SD, stable disease

A serial tumor progression diagram in patients assessed as SD initially of the low-risk and high-risk groups stratified by risk-scoring system was demonstrated in Fig. 6. Finally, 18 and 3 patients in the low-risk and high-risk groups achieved no disease progression after 12 months, respectively.

## Discussion

In the present study, the ECV fraction derived from PVP was proven to be an independent risk factor for progression in LAPC patients assessed with SD initially after IORT. Moreover, a risk-scoring system integrating ECV, CT radiological features, and CA19-9 response as a novel biomarker was constructed, and utilized to predict PFS in LAPC patients assessed with SD, which could further stratify the risk of progression in these patients with satisfactory prognostic predictive performance. Our scoring system could serve as a complement to the RECIST v.1.1 criteria to identify LAPC patients with SD who would develop progress at risk after IORT, allowing clinicians to adopt appropriate treatment strategies and improve the prognosis.

In this study, 103 patients included 64 males and 39 females, with a mean age of 58.52 ± 10.09 years, which is similar to the previous study [25]. Demographic breakdown in our study aligns with known characteristics of LAPC patients.

Given the fact that PDAC is rich in stromal components, the treatment-induced desmoplastic stromal response, edema, and inflammatory response after radiotherapy can lead to no apparent change in lesion diameter or even pseudo-progression [26]. As a result, evaluating and reflecting true therapeutic benefits, and stratifying the prognosis based on conventional morphological changes in SD patients are challenging. Various risk stratification methods have already been reported to overcome the drawbacks of RECIST in PDAC [9–11, 27]. Yang et al proposed a radiomics signature to predict outcomes in LAPC patients with SD [11]. Another study revealed that lower circulating tumor DNA before treatment was associated with SD [27]. However, the clinical application of these methods was limited due to their complexity, poor repeatability, time-consuming nature, or high cost. Consequently, it is crucial to investigate a new simple, and effective approach for progression risk stratification in SD patients to facilitate precise therapeutic decisions. The four predictors identified in our risk-scoring system were easily accessible and routinely used in the clinic.

Our study showed the high value of ECV derived from PVP indicated a low risk of progression after IORT. This could be explained by the fact that PDAC contains a lot of tumor stroma and a poor blood supply [7]. ECV has been shown to be associated with fibrosis, desmoplastic stroma, and tissue elasticity [18]. A reduced ECV value indicates vascular deficiency and severe necrosis induced by hypoxia. Furthermore, tumor hypoxia contributes to the sensitivity of radiotherapy, which results in radiotherapy resistance and a poor prognosis [28, 29]. In contrast, a high ECV reflects an enlarged extracellular space that might be composed of abundant micro-vessels at the histopathological level, potentially resulting in a low level of hypoxia and thus more sensitivity to radiotherapy. The expansion of extracellular space facilitates the penetration and distribution of chemotherapy drugs, allowing adjuvant chemotherapy to kill tumor cells more effectively. All the above reasons might contribute to the relatively good prognosis in patients with high ECV. In this study, we adopted ECV fraction derived from PVP rather than other delayed times (3, 5, or 10 min) [18, 30], which effectively reduced the examination time and radiation doses. Moreover, there were also articles that calculated ECV late-arterial-phase [31]. At present, there is no consensus on the delay time for calculating ECV. PVP, one of the conventional contrast-enhanced phases in pancreatic protocol CT, is routinely used in clinical practice and it might be a potential alternative to the equilibrium phase for calculating ECV [14]. Meanwhile, no attempt has been made to generate ECV fraction by PVP previously. Although Noid calculated ECV fraction based on late-arterial-phase, they only analyzed the association between ECV and CA19-9 and did not directly investigate its correlation with treatment response [31]. Our study confirmed that ECV in PVP was an independent predictor of prognosis.

Two CT radiological features, rim enhancement, and peripancreatic fat infiltration, were demonstrated as predictors for the progression of LAPC in our study, as previously reported [21, 32]. It has been revealed that the hypo-attenuation areas in rim-enhancement were significantly associated with high histological grade, rapid proliferation, few residual acini, and severe necrosis [21, 33], which might result in tumor hypoxia, consequently leading to resistance to radiotherapy. From this perspective, the presence of rim-enhancement might indicate insensitivity to radiotherapy [28, 29]. Recently, some studies have found that peripancreatic fat infiltration, which reflects the extent of tumor invasion to surrounding tissues, is associated with a low R0 resection rate and poor prognosis [12, 32]. These are consistent with the findings of our study.

Serum CA19-9 is a widely used tumor marker, treatment response indicator, and prognostic predictor in PDAC [14, 34, 35]. CA19-9 positively correlated with tumor load, and a decline over 20%, 50%, or to normality after treatment was reported to be an indicator of favorable prognosis [34, 36]. A recent clinical trial utilized a 50% reduction in CA19-9 levels after treatment as one of the criteria for evaluating response to treatment [35], and Newhook et al had previously demonstrated that a decrease of CA19-9 levels over 50% or to normal implies improved overall survival (OS) [34, 36]. What’s more, the normal range of serum CA19-9 was less than 37 U/mL according to the NCCN guideline for PDAC [14]. However, as the literature reported, there exist CA19-9 non-secretors with very low CA19-9 levels (≤ 2 U/mL) or normal ranges (< 37 U/mL) [37, 38]. To avoid difficult or inaccurate assessment of patients with normal CA19-9 levels who had a relatively small decrease in CA19-9 or remained within the normal range, we only included patients with CA19-9 > 37 U/mL. Therefore, our study adopted the criteria of a decrease of over 50% or to a normal range for CA19-9 responders, which confirmed a longer PFS time compared with non-responders.

Our study had several limitations. First, the sample size was relatively small, and the patients were from a single institution and a retrospective cohort. Thus, a prospective cohort from multi-centers is required to validate the results of this study. Second, multiple CT scanners were used due to the retrospective study. A standardized scanning parameter and contrast injection protocols were followed for adopted for all patients in this study, which might minimize the variations between devices and ensure comparable imaging results. Third, we only included LAPC patients with CA19-9 over 37 U/mL, so the scoring system is not applicable to all pancreatic cancer patients, such as Lewis negative PDAC. Fourth, only PFS, but not OS was analyzed in the present study. Fifth, the consistency or correlation between PVP-based ECV and equilibrium-based ECV was not investigated because of the retrospective nature of this study. Prospective research should be performed to evaluate whether PVP-based ECV could replace equilibrium-based ECV in the future. Last, CT-based ECVs are not suitable for patients with CECT contraindications, such as iodine allergy. Whether magnetic resonance imaging-based ECV could replace CT-based ECV remains to be investigated. Therefore, further research is needed to address the above concerns.

In conclusion, ECV derived from PVP can be used in predicting the progression risk in LAPC patients initially assessed as SD after IORT. The risk-scoring system integrating ECV, CT radiological features, and CA19-9 response could serve as an efficient and practical tool for prognosis stratification in LAPC patients with SD. It could assist RECIST v.1.1 to further identify SD patients who might be sensitive to and benefit from IORT accurately, aiding clinicians in choosing individual treatment strategies, preventing tumor progression, and improving the prognosis of patients with LAPC after IORT.

### Supplementary information

Supplementary material

## Data Availability

The datasets generated during and/or analyzed during the current study are available from the corresponding author upon reasonable request.

## Abbreviations

AJCC: American Joint Committee on Cancer
AP: Arterial phase
CA 242: Carbohydrate antigen 242
CA19-9: Carbohydrate antigen 19-9
CEA: Carcinoembryonic antigen
CECT: Contrast-enhanced CT
CI: Confidence interval
CT: Computed tomography
ECV: Extracellular volume
HR: Hazard ratio
IORT: Intraoperative radiotherapy
LAPC: Locally advanced pancreatic cancer
NCCN: National Comprehensive Cancer Network
PDAC: Pancreatic ductal adenocarcinoma
PFS: Progression-free survival
PPP: Pancreatic parenchymal phase
PVP: Portal venous phase
RECIST: Response evaluation criteria in solid tumor
ROC: Receiver operating characteristic
SD: Stable disease
VOI: Volume of interest

## References

[1] Suker M, Beumer BR, Sadot E (2016). FOLFIRINOX for locally advanced pancreatic cancer: a systematic review and patient-level meta-analysis. Lancet Oncol.

[2] Macedo FI, Ryon E, Maithel SK (2019). Survival outcomes associated with clinical and pathological response following neoadjuvant FOLFIRINOX or gemcitabine/nab-paclitaxel chemotherapy in resected pancreatic cancer. Ann Surg.

[3] Gutt R, Liauw SL, Weichselbaum RR (2010). The role of radiotherapy in locally advanced pancreatic carcinoma. Nat Rev Gastroenterol Hepatol.

[4] Li Y, Feng Q, Jin J (2019). Experts’ consensus on intraoperative radiotherapy for pancreatic cancer. Cancer Lett.

[5] Calvo FA, Krengli M, Asencio JM (2020). ESTRO IORT task force/ACROP recommendations for intraoperative radiation therapy in unresected pancreatic cancer. Radiother Oncol.

[6] Hester CA, Perri G, Prakash LR (2022). Radiographic and serologic response to first-line chemotherapy in unresected localized pancreatic cancer. J Natl Compr Canc Netw.

[7] Perri G, Prakash L, Wang H (2021). Radiographic and serologic predictors of pathologic major response to preoperative therapy for pancreatic cancer. Ann Surg.

[8] Philip PA, Lacy J, Portales F (2020). Nab-paclitaxel plus gemcitabine in patients with locally advanced pancreatic cancer (LAPACT): a multicentre, open-label phase 2 study. Lancet Gastroenterol Hepatol.

[9] Kim SS, Lee S, Lee HS, Bang S, Han K, Park MS (2022). Retrospective evaluation of treatment response in patients with nonmetastatic pancreatic cancer using CT and CA 19-9. Radiology.

[10] Meijer LL, Garajova I, Caparello C (2020). Plasma miR-181a-5p downregulation predicts response and improved survival after FOLFIRINOX in pancreatic ductal adenocarcinoma. Ann Surg.

[11] Yang Q, Mao Y, Xie H (2022). Identifying outcomes of patients with advanced pancreatic adenocarcinoma and RECIST stable disease using radiomics analysis. JCO Precis Oncol.

[12] Kim DW, Lee SS, Kim SO (2020). Estimating recurrence after upfront surgery in patients with resectable pancreatic ductal adenocarcinoma by using pancreatic CT: development and validation of a risk score. Radiology.

[13] Park SJ, Kim JH, Joo I, Lee KB, Han JK (2021). Important CT and histopathological findings for recurrence and overall survival in patients with pancreatic ductal adenocarcinoma who underwent surgery after neoadjuvant FOLFIRINOX. Eur Radiol.

[14] Tempero MA, Malafa MP, Al-Hawary M (2021). Pancreatic adenocarcinoma, version 2.2021, NCCN clinical practice guidelines in oncology. J Natl Compr Canc Netw.

[15] Tirkes T, Yadav D, Conwell DL (2022). Quantitative MRI of chronic pancreatitis: results from a multi-institutional prospective study, magnetic resonance imaging as a non-invasive method for assessment of pancreatic fibrosis (MINIMAP). Abdom Radiol (NY).

[16] Nieskoski MD, Marra K, Gunn JR (2017). Collagen complexity spatially defines microregions of total tissue pressure in pancreatic cancer. Sci Rep.

[17] Carvalho TMA, Di Molfetta D, Greco MR (2021). Tumor microenvironment features and chemoresistance in pancreatic ductal adenocarcinoma: insights into targeting physicochemical barriers and metabolism as therapeutic approaches. Cancers (Basel).

[18] Wang ZJ, Zhang TT, An C (2020). Estimation of fractional extracellular space at ct for predicting chemotherapy response and survival in pancreatic ductal adenocarcinoma. AJR Am J Roentgenol.

[19] Tanaka M, Heckler M, Mihaljevic AL (2023). Induction chemotherapy with FOLFIRINOX for locally advanced pancreatic cancer: a simple scoring system to predict effect and prognosis. Ann Surg Oncol.

[20] Al-Hawary MMFI, Chari ST, Fishman EK (2014). Pancreatic ductal adenocarcinoma radiology reporting template: consensus statement of the society of abdominal radiology and the american pancreatic association. Gastroenterology.

[21] Lee S, Kim SH, Park HK, Jang KT, Hwang JA, Kim S (2018). Pancreatic ductal adenocarcinoma: rim enhancement at MR imaging predicts prognosis after curative resection. Radiology.

[22] Elbanna KY, Jang HJ, Kim TK (2020). Imaging diagnosis and staging of pancreatic ductal adenocarcinoma: a comprehensive review. Insights Imaging.

[23] Sullivan LM, Massaro JM, D’Agostino RB (2004). Presentation of multivariate data for clinical use: the framingham study risk score functions. Stat Med.

[24] Ingrisch M, Schneider MJ, Norenberg D (2017). Radiomic analysis reveals prognostic information in T1-weighted baseline magnetic resonance imaging in patients with glioblastoma. Invest Radiol.

[25] Park W, Chawla A, O’Reilly EM (2021). Pancreatic cancer: a review. JAMA.

[26] Zins M, Matos C, Cassinotto C (2018). Pancreatic adenocarcinoma staging in the era of preoperative chemotherapy and radiation therapy. Radiology.

[27] Tjensvoll K, Lapin M, Buhl T (2016). Clinical relevance of circulating KRAS mutated DNA in plasma from patients with advanced pancreatic cancer. Mol Oncol.

[28] Buckley AM, Lynam-Lennon N, O’Neill H, O’Sullivan J (2020). Targeting hallmarks of cancer to enhance radiosensitivity in gastrointestinal cancers. Nat Rev Gastroenterol Hepatol.

[29] Tao J, Yang G, Zhou W (2021). Targeting hypoxic tumor microenvironment in pancreatic cancer. J Hematol Oncol.

[30] Fukukura Y, Kumagae Y, Higashi R (2019). Estimation of extracellular volume fraction with routine multiphasic pancreatic computed tomography to predict the survival of patients with stage iv pancreatic ductal adenocarcinoma. Pancreas.

[31] Noid G, Godfrey G, Hall W (2023). Predicting treatment response from extracellular volume fraction for chemoradiation therapy of pancreatic cancer. Int J Radiat Oncol Biol Phys.

[32] Safi SA, Haeberle L, Heuveldop S (2021). Pre-operative MDCT staging predicts mesopancreatic fat infiltration—A novel marker for neoadjuvant treatment?. Cancers (Basel).

[33] Hattori YGT, Zen Y, Mochizuki K, Kitagawa H, Matsui O (2010). Poorly enhanced areas of pancreatic adenocarcinomas on late-phase dynamic computed tomography: comparison with pathological findings. Pancreas.

[34] Newhook TE, Vreeland TJ, Griffin JF (2023). Prognosis associated with CA19-9 response dynamics and normalization during neoadjuvant therapy in resected pancreatic adenocarcinoma. Ann Surg.

[35] Ozaka M, Nakachi K, Kobayashi S (2023). A randomised phase II study of modified FOLFIRINOX versus gemcitabine plus nab-paclitaxel for locally advanced pancreatic cancer (JCOG1407). Eur J Cancer.

[36] Boone BA, Steve J, Zenati MS (2014). Serum CA 19-9 response to neoadjuvant therapy is associated with outcome in pancreatic adenocarcinoma. Ann Surg Oncol.

[37] Bauer TM, El-Rayes BF, Li X (2013). Carbohydrate antigen 19-9 is a prognostic and predictive biomarker in patients with advanced pancreatic cancer who receive gemcitabine-containing chemotherapy: a pooled analysis of 6 prospective trials. Cancer.

[38] Luo G, Fan Z, Cheng H (2018). New observations on the utility of CA19-9 as a biomarker in Lewis negative patients with pancreatic cancer. Pancreatology.

