# Supplementary material for: Extracellular volume-based scoring system for tracking tumor progression in pancreatic cancer patients receiving intraoperative radiotherapy
Source: Insights Imaging. 2024 May 12;15:116. doi: 10.1186/s13244-024-01689-6 (PMC11089023; doi:10.1186/s13244-024-01689-6)
Supplement: Supplementary file 1 — Supplementary material [file 13244_2024_1689_MOESM1_ESM.pdf]

Extracellular volume-based scoring system for tracking tumor progression in pancreatic cancer patients receiving intraoperative radiotherapy

ELECTRONIC SUPPLEMENTARY MATERIAL

1. Materials and methods

1.1 Appendix E1: Steps to ensure consistency in delineation

To ensure consistency in delineation, we have carried out the following steps：

1. Clear criteria: Develop standardized definitions of radiological features and provide images of typical signs help readers to evaluate radiological features more consistently. These criteria should be viewed as a guide to ensure that readers reach a common understanding of the lesion and reduce the likelihood of interpretation discrepancies.

2. Training and examination before assessment： Provide ongoing training and education for all readers to ensure that they know and understand the criteria for lesion assessment. Training can include demonstrations and evaluation of actual cases in advance.

3. Identification of inconsistencies： The third professor and two readers read the images together to resolve inconsistencies.

These steps help to establish a consistent, standardized process for tumor evaluation, thereby ensuring consistency among readers and improving the reliability and comparability of results.

1. Materials and methods

1.2 Appendix E2: IORT and adjuvant therapy

A multidisciplinary team, which included surgeons, radiation oncologists, and radiologists, determined the appropriate treatment strategy of IORT and the following adjuvant therapy in LAPC patients. During surgery, a cytological diagnosis and a gross examination were routinely performed to confirm the diagnosis of pancreatic cancer without peritoneal or liver metastases. Before IORT, palliative surgery, including gastrojejunostomy or biliary bypass as appropriate, was regularly carried out depending on the tumor's location and clinical symptoms. After palliative surgery, the extent of lesion at operation was accessed by the surgeon and radiation oncologist, then a field including the pancreatic mass with a 0.5-1.0cm margin around and regional lymph nodes was covered by a cone [1]. The electron energy of electron beam employed in IORT was 9-12 Mev depending on the maximum diameter of tumor, with an average dose of 14.8 Gy (range 13.5-15.0 Gy). In order to protect adjacent normal tissue, the IORT field excluded the liver, gastric wall and small bowels.

Sequential adjuvant therapy was composed of chemotherapy or chemoradiotherapy according to abdominal oncology multidisciplinary team.

Chemoradiotherapy regimen: Intensity modulated radiation therapy (IMRT) was used. 95% planning gross tumor volume (PGTV) received a total radiation dose of 45–54 Gy and 1.8–2 Gy each fraction for 28 fractions over 4 weeks. The patients were treated with concurrent gemcitabine (GEM) (at a dose of 1000mg/m^2 for 3 of every 4weeks).

Chemotherapy regimen: Patients in our study were all in locally advanced stage and were in poor physical condition so they could not tolerate FOLFIRINOX or modified FOLFIRINOX. Accordingly, chemotherapy regimens only include gemcitabine-based regimens (GEM, GEM plus albumin-bound paclitaxel, or GEM plus S-1) (2–6 cycles). The final regimen was determined by multidisciplinary team according to the stage and physical condition of patients. The detailed regimens were as follows:

- GEM plus albumin-bound paclitaxel: albumin-bound paclitaxel at 125 mg/m^2 and gemcitabine at 1000 mg/m^2 on days 1,8 and 15, every four weeks.

- GEM plus S-1: infusion gemcitabine at 1000 mg/m^2 on day 1 and day 8, taken orally twice of S-1 at 60–100 mg/day on day 1–14, every 3 weeks.
- GEM: infusion at 1000 mg/m^2 weekly to 7 weeks and rest for 1 week, then continuous for 3 weeks and rest for 1 week, every four weeks.

Details on the number of patients in different treatment modalities was shown in Table S4.

References:

[1] Li Y, Feng Q, Jin J, et al. Experts' consensus on intraoperative radiotherapy for pancreatic cancer. Cancer Lett 2019;449:1-7. doi: 10.1016/j.canlet.2019.01.038.

2. Supplementary tables

2.1 Table S1. Multiphase contrast-enhanced CT scan parameter

Tube current was adjusted using automatic tube current modulation

	Revolution CT	Discovery CT 750 HD	Lightspeed VCT	Optima CT 660
No. of channels	256	64	64	64
Section collimation*	128 × 0.625	64 × 0.625	64 × 0.625	64 × 0.625
Thickness(mm)	1.25	1.25	1.25	1.25
Interval(nm)	5	5	5	5
Helical pitch	0.992	0.984	0.984	1.375
Gantry rotation time(s)	0.5	0.7	0.5	0.5
Tube voltage (kVp)	120	120	120	120
Matrix	512 × 512	512 × 512	512 × 512	512 × 512

*Number of detector rows times section thickness (mm)

2.2 Table S2. Definition of each CT imaging feature in this study

CT radiological feature	Definitions	
**Necrosis**	Tumoral tissue that did not enhance on multiphasic CT examinations after contrast medium injection	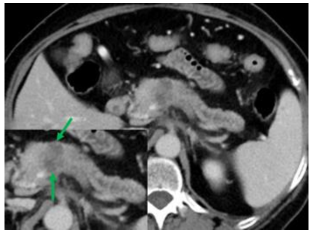
**Rim-enhancement**	irregular ringlike enhancement with a relatively hypo-vascular central area on dynamic-enhanced images	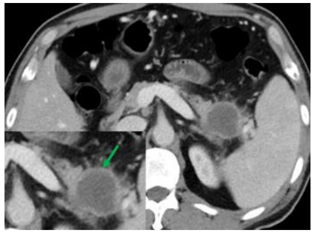
**Peripancreatic fat infiltration**	Tumor directly attached to the peri-pancreatic adipose tissues or extending directly from the intra-pancreatic tumor to the extra-pancreatic adipose tissues resulting in blurring or hyperdense shadowing at the interface between the tumor and the adipose tissues	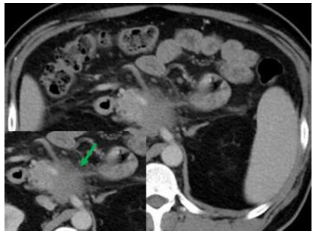
**Suspicious lymph nodes**	Including any of the following: short axis > 1 cm, abnormal round morphology, heterogeneity, or central necrosis.	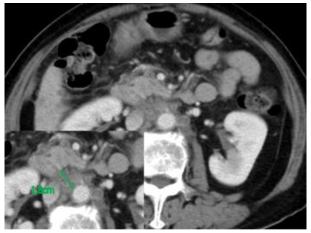

**Pancreatic duct dilatation**	Interrupted main pancreatic duct was defined as an abrupt luminal disruption of the main pancreatic duct.	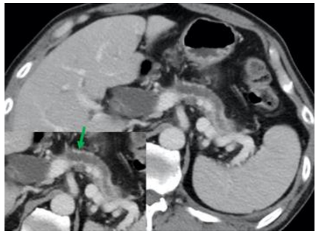
**Atrophic upstream pancreatic parenchyma**	A main pancreatic duct calibre to total pancreatic parenchymal width ratio of less than 0.50.	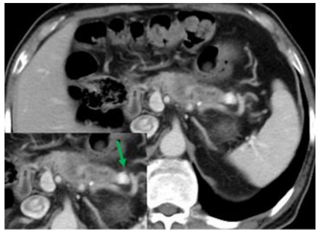

References:[1-4]

1.Lee S, Kim SH, Park HK, et al. Pancreatic Ductal Adenocarcinoma: Rim Enhancement at MR Imaging Predicts Prognosis after Curative Resection. *Radiology* 2018;288(2):456-66. doi: 10.1148/radiol.2018172331.

2.Tamada T, Ito K, Kanomata N, et al. Pancreatic adenocarcinomas without secondary signs on multiphasic multidetector CT: association with clinical and histopathologic features. *Eur Radiol* 2016;26(3):646-55. doi: 10.1007/s00330-015-3880-3.

3.AlHawary MM FI, Chari ST, Fishman EK, Hough DM, Lu DS, Macari M, Megibow AJ, Miller FH, Mortele KJ, Merchant NB, Minter RM, Tamm EP, Sahani DV, Simeone DM. . Pancreatic ductal adenocarcinoma radiology reporting template: consensus statement of the society of abdominal radiology and the american pancreatic association. *Gastroenterology* 2014;146(1):291-304. doi: 10.1053/j.gastro.2013.11.0042.

4. Yoon SH LJ, Cho JY, Lee KB, Kim JE, Moon SK, Kim SJ, Baek JH, Kim SH, Kim SH, Lee JY, Han JK, Choi BI. Small (≤ 20 mm) pancreatic adenocarcinomas: analysis of enhancement patterns and secondary signs with multiphasic multidetector CT. *Radiology* 2011;259:442–52. doi: 10.1148/radiol.11101133

2.3 Table S3. Explanations for the selection of clinical parameters in this study

Clinical parameters	Reasons	References
**AJCC 8th TNM stage**	TNM staging is basic information, which can be used to guide practical treatment planning and assess prognosis[1]	[1]Park W, Chawla A, O'Reilly EM. Pancreatic Cancer: A Review. *JAMA* 2021;326(9):851-62.
**Jaundice/TBil/DBil**	Obstructive jaundice is one of the most frequent clinical problems of PDAC and it influence chemotherapy completion and survival in patients with advanced PDAC. Jaundice and bile duct obstruction often cause hyper-bilirubinemia [2].	[2]Gasparini G, Aleotti F, Palucci M, et al. The role of biliary events in treatment and survival of patients with advanced pancreatic ductal adenocarcinoma. *Dig Liver Dis* 2023;55(12):1750-56.
**CA19-9/ CEA/CA242**	As to tumor biomarkers, CA19-9 has been validated as an essential biomarker for PDAC [3]. Meanwhile, serum CEA also an independent prognostic factor, combining the two markers significantly improved the prognostic accuracy of CA19-9 [4 5].	[3]Pancreatic Adenocarcinoma, Version 1.2022, NCCN Clinical Practice Guidelines in Oncology. *Journal of the National Comprehensive Cancer Network* 2022<br>[4]Liang Y, Cui J, Ding F, et al. A new staging system for postoperative prognostication in pancreatic ductal adenocarcinoma. *iScience* 2023;26(9):107589.<br>[5]Liu L, Xu H, Wang W, et al. A preoperative serum signature of CEA+/CA125+/CA19-9 >/= 1000 U/mL

		indicates poor outcome to pancreatectomy for pancreatic cancer. *Int J Cancer* 2015;136(9):2216-27.
**BMI /Albumin**	BMI and Serum albumin reflect the nutritional status of PDAC, with lower levels predicting a poorer prognosis [6]	[6]Riner AN, Herremans KM, Vudatha V, et al. Heterogeneity of weight loss and transcriptomic signatures in pancreatic ductal adenocarcinoma. *J Cachexia Sarcopenia Muscle* 2023 doi: 10.1002/jcsm.13390. Epub ahead of print.
**Glucose**	Since diabetes is a known risk factor for pancreatic cancer and glucose is necessary for the diagnosis of diabetes, glucose was included [7]	[7]Khadka R, Tian W, Hao X, et al. Risk factor, early diagnosis and overall survival on outcome of association between pancreatic cancer and diabetes mellitus: Changes and advances, a review. *Int J Surg* 2018;52:342-46.
**D-dimer**	Elevated D-dimer levels independently predicted poorer OS in PDAC patients[8].	[8]Chen H, Li F, Zou S, et al. Preoperative plasma D-dimer independently predicts survival in patients with pancreatic ductal adenocarcinoma undergoing radical resection. World J Surg Oncol 2021;19(1):166.
**Fibrinogen**	Hyperfibrinogen is associated with the systemic inflammatory	[9]Qi Q GY, Sun M, Chen H, Wang P, Chen Z.

	response and predicts poor prognosis for advanced pancreatic cancer [9].	Hyperfibrinogen Is Associated With the Systemic Inflammatory Response and Predicts Poor Prognosis in Advanced Pancreatic Cancer. Pancreas 2015;2015 Aug(6):977-82.
**Hematocrit**	In order to calculate ECV, hematocrit was included in this study [10].	[10] Fujita N, Ushijima Y, Itoyama M, et al. Extracellular volume fraction determined by dual-layer spectral detector CT: Possible role in predicting the efficacy of preoperative neoadjuvant chemotherapy in pancreatic ductal adenocarcinoma. Eur J Radiol 2023;162:110756.
**Transferrin**	Transferrin used to assess nutritional status in PDAC patients [11].	[11] Park JW, Jang JY, Kim EJ, et al. Effects of pancreatectomy on nutritional state, pancreatic function and quality of life. Br J Surg 2013;100(8):1064-70.

AJCC, American Joint Committee on Cancer; ALB, albumin.; BMI, body mass index; CA19-9, carbohydrate antigen 19-9; CEA, carcinoembryonic antigen; CA 242, cancer antigen 242; TBil, total bilirubin; DBil, direct bilirubin; ECV, tumor extracellular volume.

2.4 Table S4. Details on the number of patients in different treatment modalities

Treatment modalities	The number of patients
Chemotherapy	65
Gemcitabine plus albumin-bound paclitaxel	25
Gemcitabine plus S-1	18
Gemcitabine	22
Chemoradiotherapy	38

2.5 Table S5. Interobserver agreement of CT radiological features between two radiologists

**CT radiological features**	**kappa coefficient (95% CI)**
Tumor location	1.00 (1.00,1.00)
Necrosis	0.82 (0.79, 0.89)
Rim-enhancement	0.79 (0.69, 0.86)
Peripancreatic fat infiltration	0.74 (0.67, 0.86)
Suspicious lymph nodes	0.70 (0.68, 0.78)
Pancreatic duct dilatation	0.78 (0.73, 0.81)
Atrophic up stream pancreatic parenchyma	0.72 (0.67, 0.78)

Data are Cohens κ coefficients; numbers in parentheses are 95% CI.

2.6 Table S6. Interobserver agreement of CT quantitative parameters value between two radiologists

**CT quantitative parameters**	**ICC (95% CI)**
Diameter (cm)	0.87 (0.82, 0.91)
ΔHU_{tumor} (HU)	0.82 (0.78, 0.85)
$\Delta HU_{tumor}/\Delta HU_{aorta}$	0.83 (0.79, 0.86)
ECV (%)	0.83 (0.79, 0.86)

Data are ICC values; numbers in parentheses are 95% CI; ICC, intraclass correlation coefficient; ECV, extracellular volume.

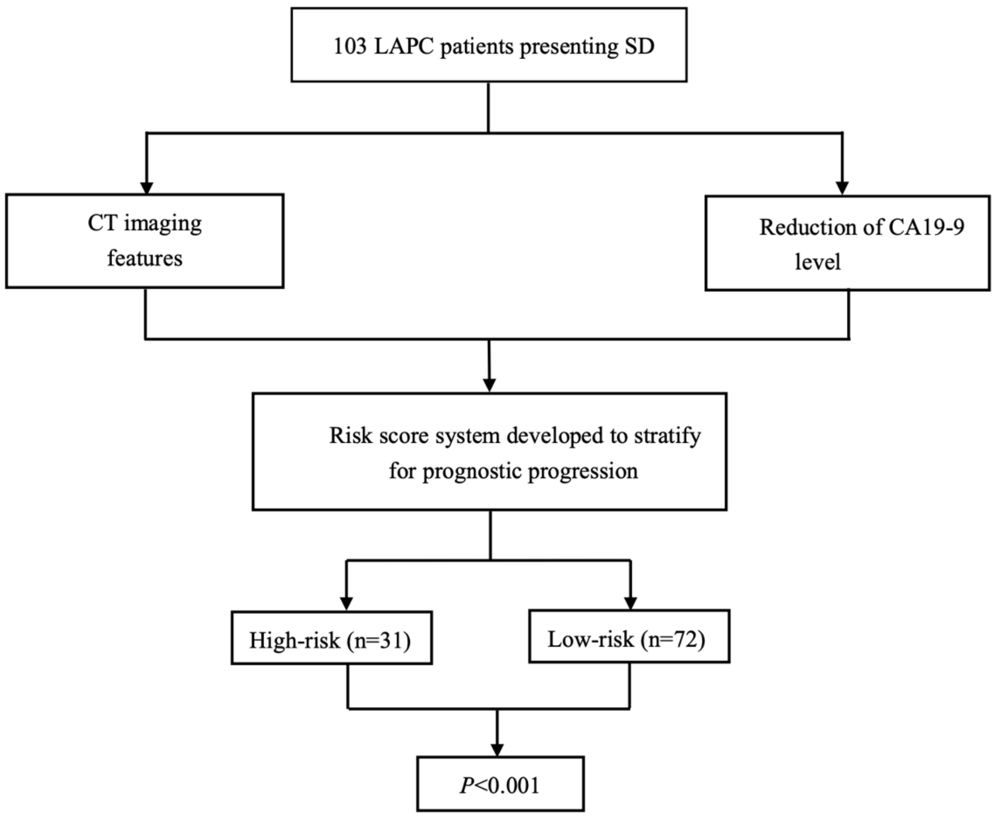

Figure S1. Illustration of this study. LAPC, locally advanced pancreatic cancer; IORT, Intraoperative radiotherapy; SD, stable disease.

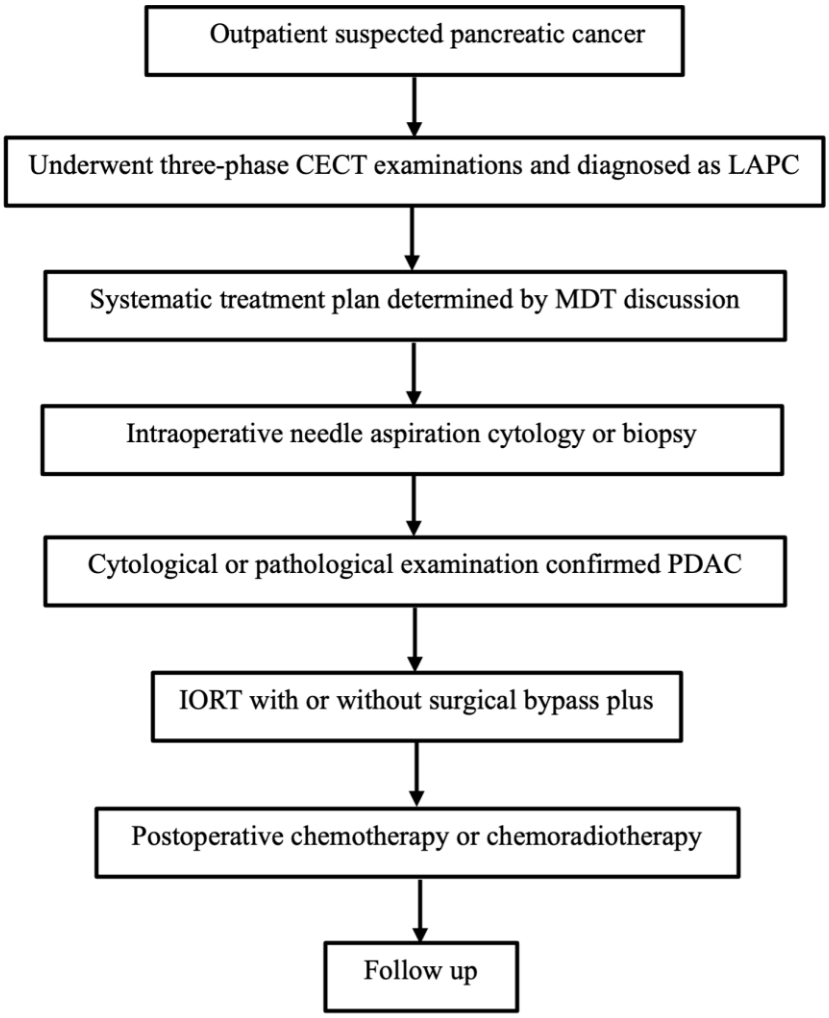

Figure S2. The illustration of IORT procedure in LAPC. IORT, intraoperative radiotherapy; LAPC, locally advanced pancreatic cancer; CECT, contrast-enhanced CT; MDT, multi-disciplinary team; PDAC, pancreatic ductal adenocarcinoma.

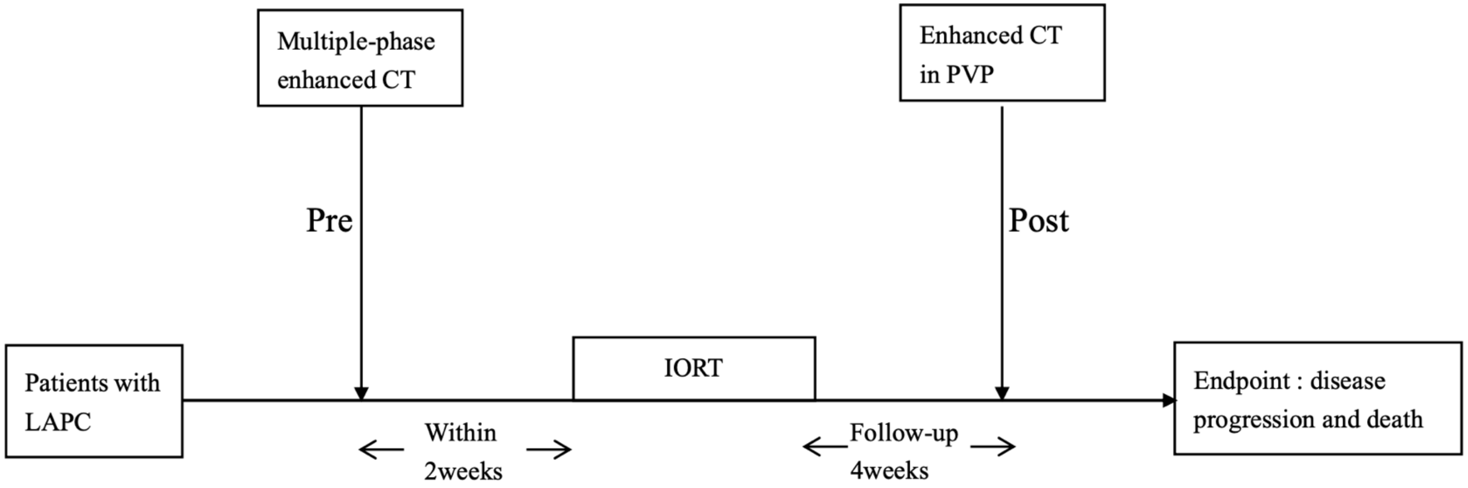

Figure S3. Schematic diagram shows treatment schedule and time plan of CT in this study. The median intervals from pre-IORT CT to IORT and was 10.0 days (range, 8.0-14.0 days).

LAPC=locally advanced pancreatic cancer, IORT= Intraoperative radiotherapy, PVP= portal venous phase.

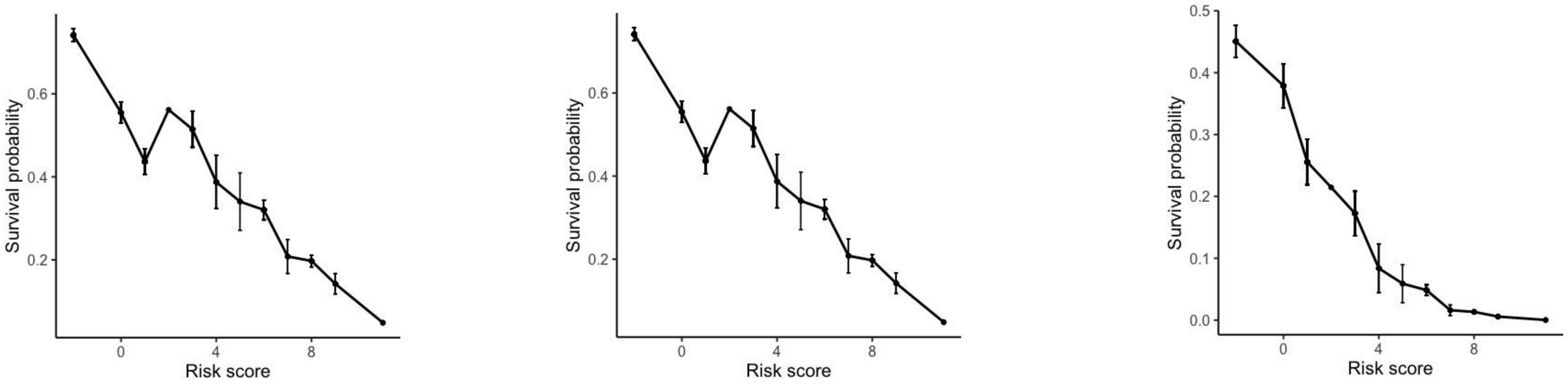

A B C

Fig S4. The progression of all LAPC patients pattern plots showing different time points for each respective risk score. **(A)** 3-month, **(B)** 6-month and **(C)** 12-month. LAPC, locally advanced pancreatic cancer.
